# Biochemical investigation of a human pathogenic mutation in the nuclear ATP5E gene using yeast as a model

**DOI:** 10.3389/fgene.2015.00159

**Published:** 2015-04-23

**Authors:** Elodie Sardin, Stéphanie Donadello, Jean-Paul di Rago, Emmanuel Tetaud

**Affiliations:** ^1^CNRS, Génétique Moléculaire des Systèmes Mitochondriaux, Institut de Biochimie et Génétique Cellulaires, UMR 5095Bordeaux, France; ^2^Université de Bordeaux, Institut de Biochimie et Génétique Cellulaires, UMR 5095Bordeaux, France

**Keywords:** ATP synthase, mitochondrial diseases, nuclear gene, yeast model, assembly

## Abstract

F_1_F_0_-ATP synthase is a key enzyme of the mitochondrial energetic metabolism responsible for the production of most cellular ATP in humans. Mayr et al. (2010) recently described a patient with a homozygote (Y12C) mutation in the nuclear gene ATP5E encoding the ε-subunit of ATP synthase. To better define how it affects ATP synthase, we have modeled this mutation in the yeast *Saccharomyces cerevisiae*. A yeast equivalent of this mutation (Y11C) had no significant effect on the growth of yeast on non-fermentable carbon sources (glycerol/ethanol or lactate), conditions under which the activity of the mitochondrial energy transducing system is absolutely essential. In addition, similar to what was observed in patient, this mutation in yeast has a minimal effect on the ATPase/synthase activities. On the contrary, this mutation which has been shown to have a strong impact on the assembly of the ATP synthase complex in humans, shows no significant impact on the assembly/stability of this complex in yeast, suggesting that biogenesis of this complex differs significantly.

## Introduction

Multi-protein complexes (I–V) embedded within the mitochondrial inner membrane are responsible for the production of cellular energy generating ATP molecules through the process of oxidative phosphorylation (OXPHOS). Defects in this process are responsible for severe diseases in humans (Dimauro, 2011; Koopman et al., 2013). The OXPHOS system has a double genetic origin: nuclear and mitochondrial. Pathogenic mutations that compromise cellular energy production can therefore be found in both the nuclear and mitochondrial genomes.

Complexes I–IV transfer electrons to oxygen coupled to a vectorial proton translocation across the mitochondrial inner membrane. The resulting transmembrane proton gradient is used by complex V, the ATP synthase, to synthesize ATP from ADP and inorganic phosphate (Boyer, 1997; Senior et al., 2002; Ackerman and Tzagoloff, 2005; Walker, 2013). The ATP synthase consists of two distinct domains, a globular matrix-localized catalytic unit called F_1_, and a membrane-embedded proton-translocating domain known as F_0_. In mitochondria, F_1_ is an assembly of five different subunits with an α_3_β_3_γδε stoichiometry, that contains three catalytic sites located in the β-subunits (Abrahams et al., 1994; Bianchet et al., 1998; Kabaleeswaran et al., 2006). The synthesis of ATP by the β-subunits depends upon rotation of the F_1_ sub-complex (called the central stalk) formed by subunits γ, δ, and ε that connects F_1_ to the proton-translocating domain (F_0_) of ATP synthase (Kabaleeswaran et al., 2006). The main components involved in proton translocation are a ring of *c*-subunits (8 in human and 10 in yeast; Stock et al., 1999; Walker, 2013) and a single subunit *a* (Stock et al., 2000; Fillingame et al., 2002). Direct contacts between the *c*-ring and subunits γ and δ enable the *c*-ring and the central stalk to rotate together as a fixed ensemble during catalysis (Stock et al., 1999; Tsunoda et al., 2001). The F_1_ is also physically connected to F_0_ via its external surface by a peripheral stalk, composed in yeast of single copies of the subunits OSCP, 4, d, h, f, 8, and i (Velours and Arselin, 2000; Ackerman and Tzagoloff, 2005; Devenish et al., 2008); this structure acts as a stator to counter the tendency of the α_3_β_3_ moiety to follow the rotation of the central stalk during catalysis. The mitochondrial ATP synthase exists as a dimer (Arnold et al., 1998), a structure mediated by subunits e and g that is important for cristae formation (Allen et al., 1989; Paumard et al., 2002). The ATPase activity of the yeast mitochondrial ATP synthase is also regulated by several peptides (If1, Stf1, and Stf2; Hong and Pedersen, 2002).

The biogenesis of ATP synthase is a particularly complex process, which involves a number of protein factors having specific actions in the expression of the mitochondrial DNA-encoded subunits and in the establishment of proper subunit interactions (Ackerman and Tzagoloff, 2005; Devenish et al., 2008; Rak et al., 2009). The ATP synthase assembly pathway has been extensively studied in *Saccharomyces cerevisiae* (Ackerman and Tzagoloff, 2005; Kucharczyk et al., 2009; Rak et al., 2009, 2011a). Although the yeast and human ATP synthases are highly similar, there must be some differences in their assembly pathways, because factors involved in the biogenesis of the yeast enzyme are not conserved in humans and *vice versa* (Cizkova et al., 2008; Houstek et al., 2009).

Diseases caused by ATP synthase defects have been associated to mitochondrial DNA mutations in the genes encoding subunits *a* and A6L (Kucharczyk et al., 2009). Autosomal recessive nuclear mutations have also been reported in genes (ATP12 and TMEM70) encoding ATP synthase assembly factors (De Meirleir et al., 2004; Cizkova et al., 2008) and structural subunits (ε and α; Mayr et al., 2010; Jonckheere et al., 2013). All these mutations share a common biochemical phenotype with a decreased content of fully assembled ATP synthase.

The ε-subunit mutation was found in a 22 years old patient presenting with neonatal onset, lactic acidosis, 3-methylglutaconic aciduria, mild mental retardation, and peripheral neuropathy (Mayr et al., 2010). This was a homozygous missense mutation replacing a highly conserved tyrosine in position 12 of the protein with cysteine. This mutation caused a substantial decrease in the rate of mitochondrial ATP synthesis apparently because of a reduced content in fully assembled ATP synthase. The authors concluded that ε-subunit has an essential role in the biosynthesis and assembly of the F_1_ domain of ATP synthase.

In the yeast *S. cerevisiae*, evidence was provided that the ε-subunit is not required for the assembly of the other subunits of ATP synthase (Tetaud et al., 2014). However, a lack in the ε-subunit rapidly results in F_0_-mediated proton leaks through the membrane, showing that the ε-subunit is in yeast essential for the coupling of the F_1_ and F_0_ domains of ATP synthase (Tetaud et al., 2014). In order to better understand how the Y12C mutation in ε-subunit affects ATP synthase, we have created a yeast model of this mutation. The results show that this mutation has only a limited impact on activity and assembly of ATP synthase in yeast.

## Materials and Methods

### Strains and Media

*Escherichia coli* NEB-5alpha strain (BioLabs) was used for the cloning and propagation of plasmids. The *S. cerevisiae* strains used and their genotypes are listed in **Table 1**. The following rich media were used for the growth of yeast: 1% (w/v) yeast extract, 1% (w/v) peptone, 40 mg/l adenine, 2% (w/v) glucose, 2% (w/v) galactose, 2% (w/v) glycerol, or 2% (w/v) lactate. The glycerol medium was buffered at pH 6.2 with 50 mM potassium phosphate, and 2% (w/v) ethanol was added after sterilization. We also used complete synthetic medium (CSM) 0.17% (w/v) yeast nitrogen base without amino acids and ammonium sulfate, 0.5% (w/v) ammonium sulfate, 2% (w/v) glucose, and 0.8% (w/v) of a mixture of amino acids and bases from Formedium. The solid media contained 2% (w/v) agar.

**Table 1:** Genotypes of yeast strains.

Name	Nuclear genotype	mtDNA	Source
YE1	*Mat*α *ade2-1 his3-11,15 leu2-3,112 trp1-1 ura3-1 arg8::HIS3 atp15::KanMX* + pCM189*-ATP15*	ρ^+^*Arg8^m^*	Tetaud et al. (2014)
Y11C	*Mat*α *ade2-1 his3-11,15 leu2-3,112 trp1-1 ura3-1 arg8::HIS3 atp15::KanMX* + pES425*-ATP15-Y11C*	ρ^+^*Arg8^m^*	This study
hATP5E	*Mat*α *ade2-1 his3-11,15 leu2-3,112 trp1-1 ura3-1 arg8::HIS3 atp15::KanMX* + pCM189*-hATP5E*	ρ^+^*Arg8^m^*	This study

### Construction of a Yeast Strain Expressing the Yeast Subunit ε (WT and Mutant Y11C)

Yeast strain (YE1) expressing WT ε-subunit was described previously (Tetaud et al., 2014). To obtain the yeast epsilon Y11C mutant, the codon TAT (31/33) was mutated to TgT to replace tyrosine 11 with cysteine. The coding sequence of the ε-subunit gene (*ATP15*) was amplified by PCR with Phusion DNA polymerase (Fermentas) using DNA from strain W303-1B as a template and the primers EY11C-5Hind (5^′^-aaaaagcttATGTCTGCCTGGAGGAAAGCTGGTATATCATgTGGCTGCATATTTG-3^′^) that include the mutation (underlined) for the sense strand and EY11C-3 (5^′^-aaagcggccgcCTATTTTGTTATTGGAGTGGGTTCAGAAGCTGCAGTGCC-3^′^) for the antisense strand. The PCR product was digested with *Hin*dIII–*Not*I and ligated into the vector pES425 (marker leucine, Doron Rapaport) to produce plasmid pES425-Y11C. The cloned gene was verified by DNA sequencing. The YE1 strain was transformed with pES425-Y11C and selected on synthetic complete medium lacking uracil and leucine. The resulting strain that contains two different plasmids (pCM189-ε WT and pES425-Y11C) was subsequently cultured for about 10 generations in a medium containing uracil allowing the lost of the pCM189-ε WT plasmid. One clone called Y11C in a ρ^+^ state and containing only pES425-Y11C plasmid was retained for further analysis.

### Construction of a Yeast Strain Expressing the Human Subunit ε (hATP5E) Under the Control of a Doxycycline-Repressible Promoter

The coding sequence of the human ε-subunit gene (*ATP5E*) was amplified by PCR using cDNA obtained from human HeLa cells as a template and primers hATP5E-5BamXho (5^′^-tttggatccctcgag**ATG**GTGGCCTACTGGAGACAGGCTGGACTCAGC-3^′^) and hATP5E-3ClaNot (5^′^-aaagcggccgcatcgat**TTA**TTCCTTCTTTACTTTCACAATTTTTACGTTG-3^′^) for the antisense strand. The PCR product was digested with *Bam*HI–*Not*I and ligated into the vector pCM189 (Gari et al., 1997) to produce plasmid pCM189-hATP5E. The cloned gene was verified by DNA sequencing. Y11C strain was transformed with pCM189-hATP5E and selected on synthetic complete medium lacking uracil and leucine. The resulting strain that contains two different plasmids (pES425-Y11C and pCM189-hATP5E) was subsequently cultured for about 5–7 generations in a medium containing leucine allowing the lost of the pES425-Y11C plasmid. One clone, hATP5E wild-type (WT) human ε, isolated from a ρ^+^ clone, and containing only pCM189-hATP5E plasmid was retained for further analysis.

### Miscellaneous Procedures

The oxygen consumption of cells was measured polarographically at 28°C using a Clark oxygen electrode in a 1 ml thermostatically controlled chamber. Respiratory rates were determined from the slope of a plot of O_2_ concentration versus time. Cell oxygen consumption was measured in fresh cultured media (CSM-galactose, OD_600_ = 2) and isolated mitochondria oxygen consumption was measured in the respiration buffer (0.65 M mannitol, 0.36 mM EGTA, 5 mM Tris-phosphate, 10 mM Tris-maleate, pH 6.8) as previously described (Rigoulet and Guerin, 1979). Isolated mitochondria were prepared by the enzymatic method (Guerin et al., 1979). The protein amounts were determined by the bicinchoninic acid assay (BCA; Smith et al., 1985) in the presence of 5% SDS. For ATP synthesis rate measurements, mitochondria (0.15 mg/ml) were placed in a 1-ml thermostatically controlled chamber at 28°C in respiration buffer. The reaction was started by the addition of 4 mM NADH and 1 mM ADP and stopped with 3.5% perchloric acid and 12.5 mM EDTA. The samples were then neutralized to pH 6.5 by the addition of KOH and 0.3 M MOPS. ATP was quantified in a luciferin/luciferase assay (PerkinElmer) with an LKB bioluminometer. The specific ATPase activity was measured at pH 8.4 by using a previously described procedure (Somlo, 1968). The activity of the F_1_F_0_-ATP synthase in ATP production was assessed by oligomycin addition (20 μg/mg of protein). BN-PAGE experiments were carried out as described previously (Schagger and von Jagow, 1991). Briefly, mitochondrial extracts solubilized with digitonin to a protein ratio of 2 g/g were separated in a 3–12% acrylamide continuous gradient gel (Invitrogen). After electrophoresis, the gel was either stained with Coomassie Blue or incubated in a solution of 5 mM ATP, 5 mM MgCl_2_, 0.05% lead nitrate, 50 mM glycine-NaOH, pH 8.4 to detect the ATPase activity (Grandier-Vazeille and Guerin, 1996). Western blot analyses were performed as previously described (Arselin et al., 1996). Polyclonal antibodies raised against yeast ATP synthase were used at a dilution of 1:50,000 for subunits Atp1; 1:10,000 for subunits Atp3 and Atp4. Monoclonal antibodies against yeast Porin and Cox2 (from Molecular Probes) were used at a dilution of 1:5,000. Nitrocellulose membranes were incubated with peroxidase-labeled antibodies at a 1:5,000 dilution (Promega), and the blot visualization was conducted with ECL reagent (Pierce).

## Results

### Heterologous Expression of the Human Epsilon Subunit in yeast

To model the ε-Y12C mutation in yeast, we first determined whether the WT human ε-subunit was able to complement yeast cells lacking the endogenous ε-subunit gene *ATP15* (Δ*atp15*, referred to as Δε). Since Δε cells have a high propensity to lose their mitochondrial genome (Tetaud et al., 2014), we proceeded as follows. WT yeast was first transformed with the yeast *ATP15* gene cloned into a plasmid under the control of a doxycycline-repressible promoter. The native chromosomal copy of *ATP15* was then deleted, yielding strain YE1 (Tetaud et al., 2014). The strain YE1 was next transformed with a plasmid containing the human ε-subunit gene (ATP5E), also under control of a doxycycline-repressible promoter, and the plasmid containing the yeast *ATP15* gene was then eliminated (see “Materials and Methods”), yielding the strain hATP5E.

**FIGURE 1:**
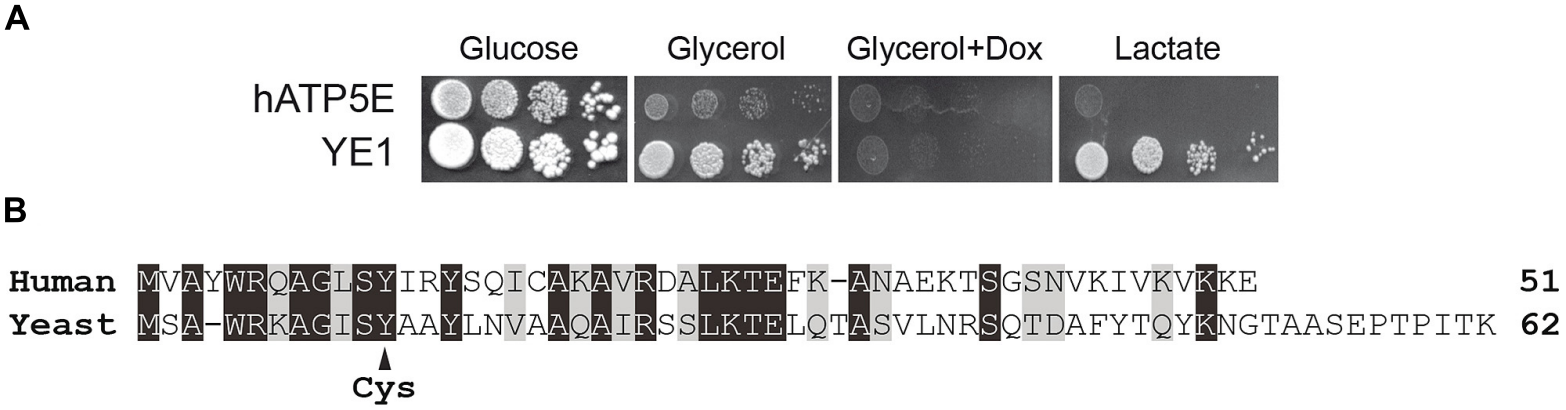
**Heterologous expression assays of human ε-subunit in yeast. (A)** Freshly grown cells of yeast expressing, human epsilon subunit (hATP5E) and WT yeast epsilon subunit (YE1) were spotted onto glucose, glycerol, and lactate media. Doxycyclin was added on plate at the concentration of 10 μg/mL. The plates were incubated at 28°C. **(B)** Sequence alignment between human and yeast ε-subunit. Identical amino acids are highlighted in black, similar amino acids in gray. Mutated Y12C in human or Y11C in yeast sequences is indicated by an arrow. Identity 37%, similarity 59%.

The hATP5E and YE1 strains grew well on fermentable carbon source (glucose, **Figure 1A**). The strain YE1 grew well also on non-fermentable carbon sources, like glycerol and lactate (**Figure 1A**). As expected, the respiratory growth of strain YE1 was abolished by blocking the expression of yeast subunit ε with doxycycline. In contrast, hATP5E cells expressing the human ε-subunit grew very poorly on respiratory medium (Glycerol or Lactate) with respect to YE1 cells (**Figure 1A**).

Although the yeast and human ε-subunit display a high level of evolutionary conservation, the two proteins show a number of amino-acids differences (**Figure 1B**). It is possible that the human protein cannot interact properly with the yeast subunits δ and γ. However, since cultures of the hATP5E strain contained only less than 5% of *petites* lacking functional mtDNA, it can be inferred that the human ε-subunit was probably assembled but functions poorly. Indeed, as we have shown, a lack in subunit ε in yeast results in massive protons that strongly destabilize the mitochondrial genome (Tetaud et al., 2014).

### Properties of Yeast Cells Expressing the Yeast ε-Subunit Carrying an Equivalent (Y11C) of the Human ε-Y12C Mutation

Due to the lack of complementation of Δε yeast with the human ε-subunit gene, we decided to investigate the consequences of the ε-Y12C mutation using the yeast subunit ε with an equivalent of this mutation. The strain YE1 was transformed with this construct, yielding strain Y11C (see “Materials and Methods”).

#### Respiratory Growth and Oxygen Consumption

**FIGURE 2:**
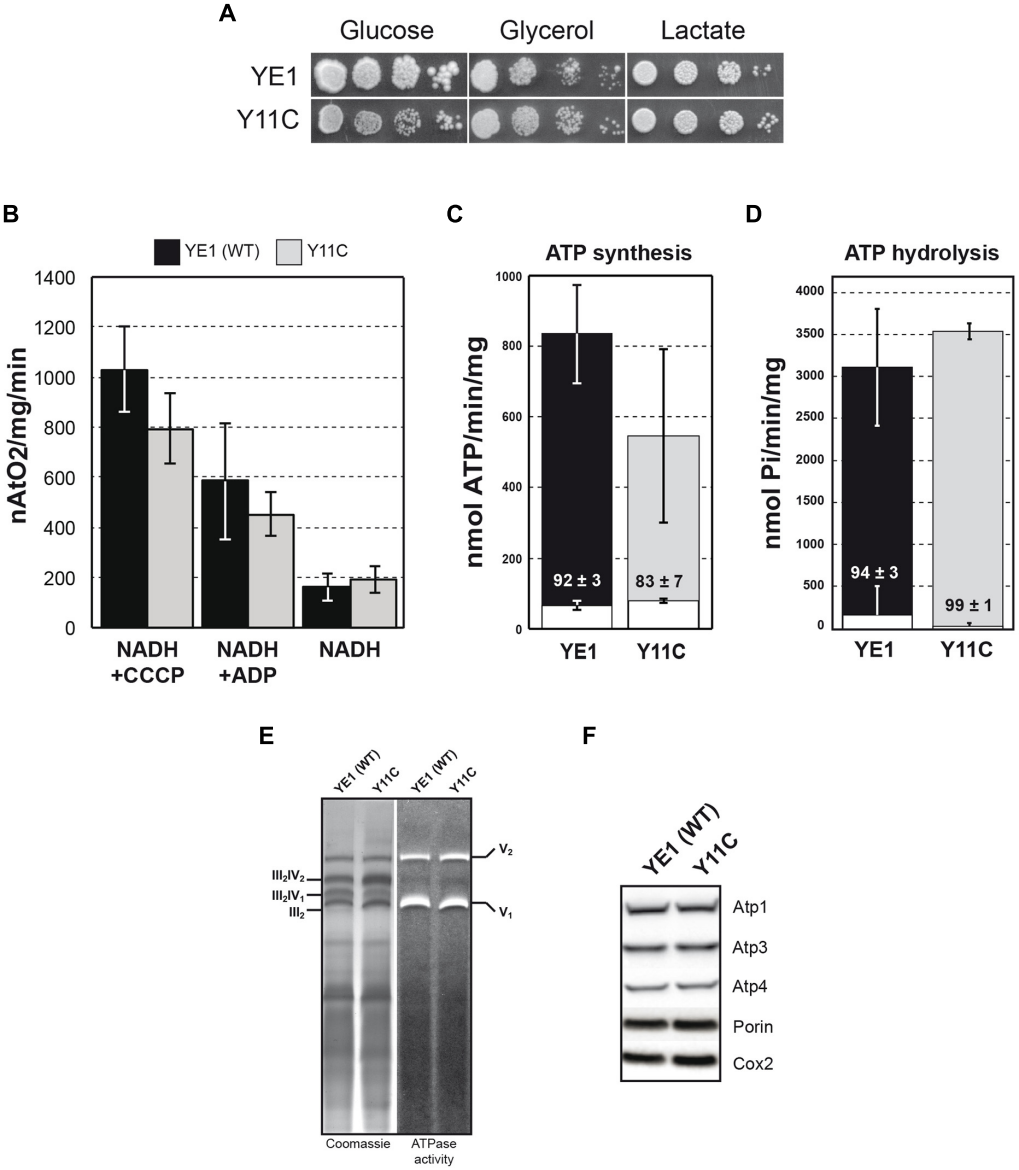
**Biochemical analysis of yeast ε-Y11C mutant. (A)** Freshly grown cells of WT (YE1) and the mutant Y11C (Y11C) were spotted onto glucose, glycerol, and lactate media. The plates were incubated at 28°C. **(B)** Oxygen consumption was measured on isolated mitochondria from WT strain YE1 and mutant Y11C grown in glycerol/ethanol 2% medium at 28°C. Additions were 0.15 mg/ml proteins, 4 mM NADH, 150 μM ADP and 4 μM CCCP (carbonyl cyanide *m*-chlorophenylhydrazone). The YE1 and Y11C cultures contained about 3–5% ρ^-/0^ cells. **(C**,**D)** Measurements of ATP synthase/ATPase activity on purified mitochondria from cells grown on glycerol/ethanol 2%. White bar in ATP synthesis/hydrolysis represent oligomycin insensitive ATPase activity and % of inhibition was indicated on the bar. The values reported are averages of triplicate assays. **(E)** BN-PAGE analyses of the ATP synthase. Mitochondria from WT (YE1) strain and yeast Y11C mutant (Y11C) from cells grown on glycerol/ethanol 2%, were solubilized with digitonin (2 g digitonin/g of protein). After centrifugation, the mitochondrial complexes were separated by BN-PAGE and the gels incubated with ATP and lead nitrate to reveal the ATPase activity (right) followed by their staining with Coomassie Blue (left). V_1_ and V_2_ correspond to monomeric and dimeric ATP synthase, respectively, and III_2_–IV_2_; III_2_–IV_1_ and III_2_ correspond to super-complexes III and IV. **(F)** Total extract proteins were separated via SDS–PAGE and probed with antibodies against the indicated proteins.

Y11C strain exhibited a nearly identically growth on both fermentable (glucose) and non-fermentable (glycerol, lactate) carbon sources (**Figure 2A**). This does not imply that the Y11C mutation has no deleterious effects, since the rate of ATP synthesis by the ATP synthase needs to be decreased by more than 70–80% in yeast to see an obvious respiratory growth defect (Mukhopadhyay et al., 1994).

We next measured the rate of oxygen consumption on isolated mitochondria. Using NADH as an electron donor, this activity was reduced by approximately 20% in mitochondria from the Y11C mutant with respect to those from the WT strain YE1, at state 3 (i.e., in the presence of an excess of external ADP, phosphorylating conditions; **Figure 2B**). With the addition of NADH alone (state 4, basal respiration) respiration was in contrast slightly increased in Y11C mitochondria in comparison to WT mitochondria, which is an indication that the passive permeability to protons of the inner membrane was increased in the mutant. Hence, the oxygen consumption rate was significantly less stimulated by ADP in the mutant than in the WT (2.4-fold versus 3.7), which indicates that the rate of ADP phosphorylation by the ATP synthase was slower in the mutant. The increased passive permeability to protons was attributed to a slight instability of the expression system used (pES425, see “Materials and Methods”), leading to an uncoupling of cells having lost the plasmid (Tetaud et al., 2014), but not necessarily an instability of ε-subunit in the ATP synthase complex.

#### Mitochondrial ATP Synthesis/Hydrolysis and ATP Synthase Assembly/Stability

The impact of the Y11C mutation on OXPHOS was further analyzed by measuring the rate of mitochondrial ATP synthesis with NADH as a respiratory substrate, in the presence of large excess of ADP (state 3). This activity was substantially reduced in the Y11C mutant by 40% compared with the WT (**Figure 2C**). As previously explained (see above), a small uncoupling will lead to a decrease in the ATP synthesis activity which does not necessarily reflect the impact of Y11C mutation. With only a 40% deficit in ATP production, it is normal that the Y11C mutant grew well on respiratory substrates, for reasons explained above.

The rate of mitochondrial ATP hydrolysis (measured on non-osmotically protected mitochondria) was not diminished, with respect to the WT, by the Y11C mutation. Furthermore, this activity was normally inhibited by oligomycin, indicating that the physical and functional coupling of F_1_ to F_0_ was mainly preserved in the mutant (**Figure 2D**). Consistent with this, the mutated F_1_F_0_-ATPase was correctly assembled and accumulated normally in Y11C cells, as shown by BN-PAGE (**Figure 2E**) and SDS-PAGE (**Figure 2F**) analyses.

## Discussion

Biochemical investigation of fibroblasts from patients carrying the ε-Y12C has revealed a substantial decrease (60%) in the accumulation of fully assembled ATP synthase with respect to control cells. Similar decreases in the rate of mitochondrial ATP synthesis and ATP hydrolysis, and in the steady-state concentrations of various subunits belonging to the F_1_ (α, β, ε) or the F_0_ (a, F6, d), were observed in the ε-Y12C cells (Mayr et al., 2010). When expression of WT ATP5E gene encoding ε-subunit was reduced, the biogenesis of ATP synthase was similarly inhibited (Havlickova et al., 2010). However, neither the ε-Y12C mutation nor a block in ε-subunit expression affects the steady-state accumulation of subunit *c*, which argues against a role of ε-subunit expression in the regulation of human ATP synthase biogenesis. Such a role has instead been ascribed to subunit *c* whose level of expression was shown to determine the steady-state concentrations of all the other subunits of the enzyme (Houstek et al., 1995; Andersson et al., 1997). Indeed, in the absence of subunit *c*, all the other ATP synthase subunits continue to be expressed at the same levels, but because of the lack in subunit *c* they cannot assemble into complete F_1_F_0_ complexes and are degraded (Houstek et al., 1995).

**FIGURE 3:**
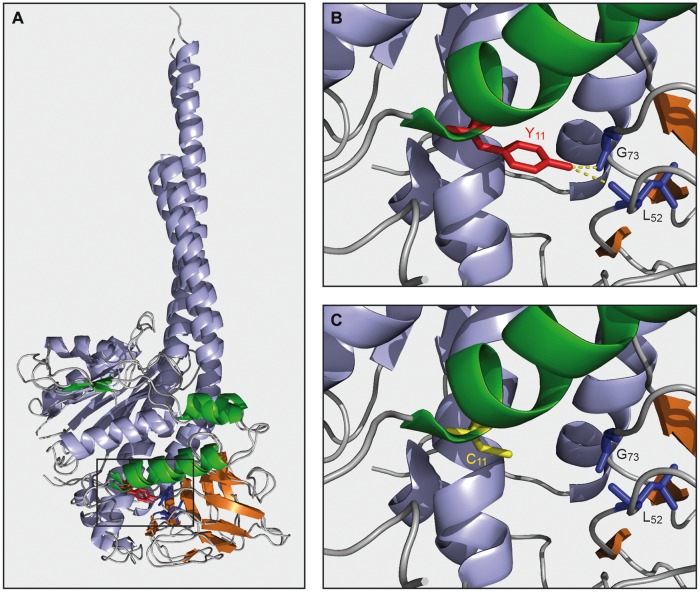
**Model of mutated ε-subunit. (A)** Alignment of γδε-subunits of bovin (2XND.pdb) and yeast (2WPD.pdb) ATP synthase built with PyMOL. γ, δ, and ε-subunits were colored in blue, orange and green, respectively. **(B)** Magnification of panel A. Yeast tyrosine (Y11, red) of ε-subunit and interaction with δ-subunit (δ-G73, δ-L52 in blue) are indicated by yellow dashed line. **(C)** Magnification of panel A and modelisation of the mutated tyrosine 11 in cysteine on the yeast ε-subunit (C11, yellow).

Studies in yeast have revealed that in the absence of ε-subunit, the other subunits of ATP synthase can still assemble. However, mitochondrial respiration is then uncoupled due to F_0_-mediated proton leaks across the mitochondrial inner membrane (Tetaud et al., 2014). It is believed that ε-subunit is required to maintain δ-subunit in the ATP synthase under the torque imposed on this subunit during rotation of the *c*-subunit ring. Upon the loss of δ-subunit, the F_0_ can no longer associate to the F_1_ and the F_0_ then dissipates the mitochondrial membrane potential. Based on the current structural models of yeast and bovine F_1_ (**Figure 3A**), there is every reason to believe that ε-subunit has a similar role in human ATP synthase. However, contrary to what happens in yeast, a functional F_0_ does not assemble in the absence of ε-subunit in humans, with notably the absence of subunit *a* where are located most of the residues involved in F_0_-mediated proton movements. Furthermore, it was shown that while unassembled F_1_ subunits aggregate in the matrix of yeast mitochondria, they are rapidly degraded in HeLa cells (Rak et al., 2011b). It can be inferred that different mechanisms regulate the expression of F_0_ in humans and yeast.

The decreased content in ε-subunit in Y12C fibroblasts is possibly due to a less efficient incorporation/stability of the mutated protein into the ATP synthase, followed by a rapid degradation of the unassembled protein. However, the present study shows that the pathogenic ε-subunit mutation (Y12C, in humans; Y11C in yeast) does not compromise the assembly of ε-subunit in yeast. Based on this finding, we propose that the lack in ε-subunit in the patient’s fibroblasts has possibly another cause than a defective assembly, like a less efficient transport of this protein into mitochondria or an increased susceptibility of the mutated protein to proteolytic degradation. It is conceivable that the ε-subunit interacts with some unknown protein until its incorporation into ATP synthase, and that this interaction is disrupted by the ε-subunit mutation making the ε-subunit more prone to degradation.

A 60% decrease in the rate of mitochondrial ATP synthesis was observed in the Y12C fibroblasts. Since, the content in fully assembled ATP synthase was decreased in a similar proportion, it was concluded that once assembled the mutated ε-subunit works like the WT protein (Mayr et al., 2010). In a similar way to humans, the ATPase/synthase activities of the yeast ATP synthase seems little affected by ε-subunit mutation, suggesting that this mutation has little or no impact on the functioning of ATP synthase. The mutated tyrosine residue establishes a few interactions with δ-subunit that are predicted to be lost by replacing this residue with cysteine (see **Figures 3B,C**). These interactions are apparently not essential for the maintenance of δ-subunit within ATP synthase. However, unlike to what has been observed in humans, the assembly of the yeast ATP synthase was not affected at all by ε-subunit mutation, suggesting differences in assembly/stability of the ATP synthase between yeast and humans.

In summary, if the ε-subunit mutation found in patients is detrimental to mitochondrial energy-transduction in humans, this mutation does not appear to have a strong impact on the yeast mitochondrial ATP synthase. The findings reported in this study point to the existence of important differences in the regulation of ATP synthase biogenesis between yeast and humans, an issue that would certainly deserve further studies for a better comprehension of how the mechanisms involved in mitochondrial biogenesis adapted during the evolution of eukaryotes.

## Author Contributions

ET designed and supervised the experiments. ES and SD performed the experiments. ET and JR analyzed the data and wrote the paper.

## Conflict of Interest Statement

The authors declare that the research was conducted in the absence of any commercial or financial relationships that could be construed as a potential conflict of interest.
